# Progression of Mild to Moderate Stenosis in the Internal Carotid Arteries of Patients With Ischemic Stroke

**DOI:** 10.3389/fneur.2018.01043

**Published:** 2018-12-03

**Authors:** Cheung-Ter Ong, Yi-Sin Wong, Sheng-Feng Sung, Chi-Shun Wu, Yung-Chu Hsu, Yu-Hsiang Su, Ling-Chien Hung

**Affiliations:** ^1^Department of Neurology Chia-Yi Christian Hospital, Chia-Yi, Taiwan; ^2^Department of Nursing, Chung Jen Junior College of Nursing Health Science and Management, Chia-Yi, Taiwan; ^3^Department of Family Medicine Chia-Yi Christian Hospital, Chia-Yi, Taiwan

**Keywords:** stroke, internal carotid artery, stenosis progression, atherosclerosis, ultrasound, low-density-lipoprotein, plaque

## Abstract

**Background and purpose:** Severe stenosis in the internal carotid artery may increase the risk of ischemic stroke. The factors that affect the progression of carotid artery stenosis in patients with ischemic stroke are poorly studied. No guidelines for the duration of follow-up of patients with ischemic stroke through carotid ultrasonography exist.

**Methods:** In this retrospective study, 179 patients (108 men; mean age, 68 years) with ischemic stroke and mild to moderate stenosis in the internal carotid artery (ICA) were recruited. Carotid artery ultrasonography was performed over the period of January 2013 to June 2016 with a median follow-up of 36 months (mean 36.5 ± 3.5 months). The severity of carotid artery stenosis was estimated with the following equation: 1– (narrowest ICA diameter/total lumen diameter at the narrowest site). The severity of stenosis was categorized into grades I (0–29%), II (30–49%), III (50–59%), and IV (60–69%). The patient's stenosis grade was defined on the basis of the stenosis rate of the ICA side with most severe stenosis.

**Results:** Stenosis progressed in 17.9% (64/358) of the vessels in 30.7% (55/179) of patients. The risk of stenosis progression increased as the severity of ICA stenosis increased. Patients with stenosis rates of above 50% are at a higher risk of stenosis progression than those with stenosis rate of < 50%. Relative to the patient group with an ICA stenosis rate of 0–29%, the adjusted odds ratios of stenosis progression were 2.33 (*p* = 0.03; 95% CI: 1.05~5.17), 3.50 (*p* = 0.09; 95% CI: 0.81~15.84), and 6.61 (*p* = 0.03; 95% CI: 1.01~39.61) in patient groups with ICA stenosis rates of 30–49%, 50–59%, and 60–69%, respectively. Hyper-LDL-cholesterolemia (Hyper-LDL-c) also increased the risk of stenosis progression, with an adjusted odds ratio of 2.22 (*p* = 0.03; 95% CI: 1.05~4.71).

**Conclusion:** The rate of ICA stenosis progression increases with stenosis grade. Patients with ICA stenosis severity >50% and Hyper-LDL-c have high rates of stenosis progression. For the patients with stroke and ICA stenosis severity >50%, annual follow up through carotid artery ultrasonography may be necessary.

## Introduction

Over the past six decades, extracranial carotid artery stenosis has been considered as a risk factor for stroke (1). Severe stenosis of the internal carotid artery (ICA) may increase the risk of stroke. Compared with patients who did not receive endarterectomy, in patients with ICA stenosis severity >70% with symptoms of stroke or transient ischemic attack (TIA), those who received endarterectomy are at a decreased risk of recurrent stroke (2, 3). In a 10-year follow-up study, Liapis et al. found that 19% of patients exhibited drastic stenosis progression in the ICA (4). Carotid stenosis is related to the formation of plaque in the carotid artery, and further development of such plaque may increase the rate of stenosis. Other factors that may increase the rate of stenosis progression in the ICA include diastolic blood pressure, diabetes mellitus, male sex, and turbulent blood flow (2, 5). Other factors that may increase the progression of carotid atherosclerosis include high plasma homocysteine levels, high plasma carotenoid levels (6), low plasma folate and vitamin B_6_ concentrations, and psychological stress. The progression of carotid artery stenosis may also be affected by the patients' lifestyles and daily activity levels (7, 8). Severe stenosis in the extracranial carotid artery is common among Taiwanese patients with ischemic stroke (9). Carotid stenosis can be estimated through noninvasive ultrasonography, which is now widely used to investigate atherosclerosis in patients with ischemic stroke. An increasing number of patients have been referred for carotid ultrasonography due to the relative ease and noninvasiveness of this examination method. Guidelines for the general management of patients with acute ischemic stroke recommend ultrasound as the first-line method for examination of the carotid artery after TIA or stroke (10). However, no guideline exists for the duration of follow-up of patients with ischemic stroke through carotid artery ultrasound. This study aims to investigate the progression rate of ICA stenosis and the factors that affect stenosis progression in patients with ischemic stroke. The adequate duration of follow-up for patients with ischemic stroke through carotid artery ultrasound was also investigated.

## Methods

### Data source

**Figure 1:**
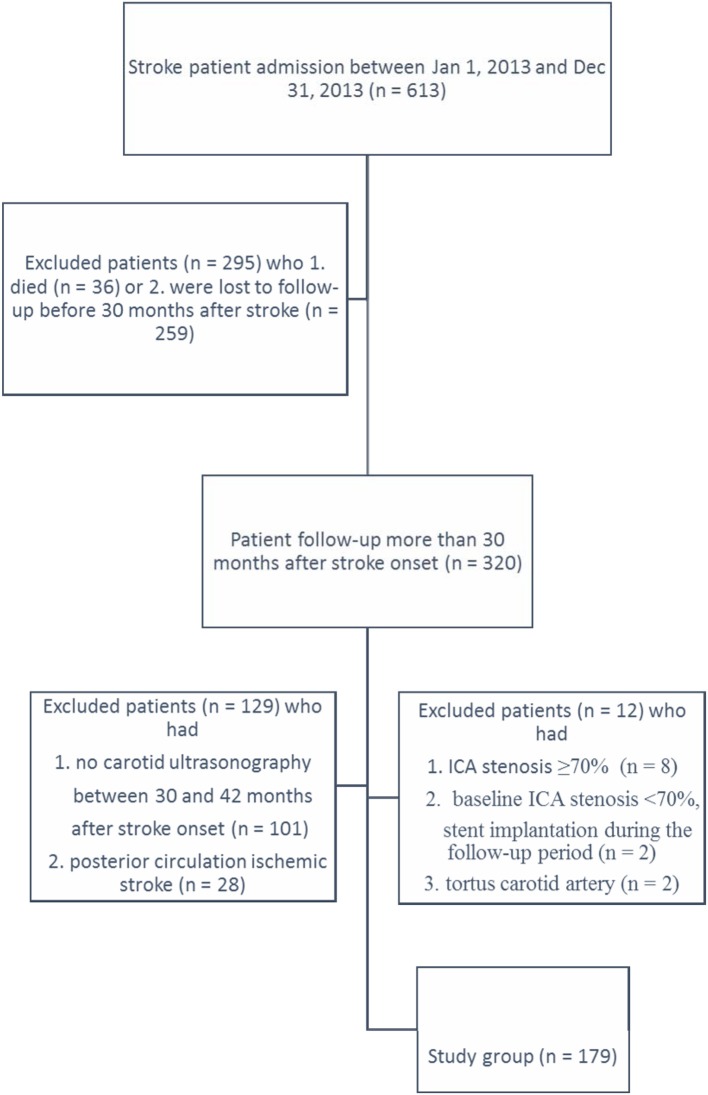
Flow chart of patient enrollment.

In this retrospective study, clinical information was collected from a stroke registration database in a single teaching hospital for patients admitted between January 1, 2013 and December 31, 2013. The hospital is an acute care, 1,000-bed teaching hospital in central Taiwan. All patients underwent baseline carotid artery ultrasonography during hospitalization for acute stroke. All patients who were followed up through carotid artery ultrasonography for 2.5–3.5 years (mean 36.5 ± 3.5 months) after stroke onset were included in this study. The following exclusion criteria were applied: 1. ICA stenosis ≥70%, 2. Receipt of carotid artery stent implantation, and 3. Tortuosity of ICA. A flow chart of patient enrollment and exclusion is shown in Figure 1. There was no significant difference in risk factors for stroke between the study participants and those were excluded from the study [hypertension (*p* = 0.2), diabetes mellitus (*p* = 1.0), hypercholesterolemia (*p* = 0.27) and hyper-LDL-cholesterolemia (*p* = 0.6)].

Hypertension was defined as systolic blood pressure ≥140 mmHg or diastolic blood pressure >90 mmHg or on the basis of a self-reported history of hypertension or use of antihypertensive agents. Diabetes mellitus was defined as pathologically elevated fasting blood sugar or on the basis of a self-reported patient history of diabetes mellitus or regular use of anti-diabetic medications. Hypercholesterolemia was defined as a total cholesterol level >200 mg/dl, hypertriglyceridemia was defined as a serum triglyceride level >160 mg/dl and hyper-LDL-c was defined as low-density-lipoprotein cholesterol (LDL) >130mg/dl (measured during acute stroke stage). Statin use was defined as patient statin possession ratio >80% (number of days of statin supply divided by the number of days in the follow up period).

### Carotid artery measures

Duplex sonographic examinations were performed in the neurophysiological laboratory of the hospital. Examinations were performed with either an Acuson sequoia or a PHILPS iE33 color duplex scanner. All examinations were performed by a well-trained technologist. The technician was blind to the previous measurement and patient's clinical details. The bilateral ICA, external carotid artery, and vertebral artery were examined through gray-scale imaging, color duplex imaging, and spectral analysis. Peak systolic blood flow velocity and end-diastolic flow velocity were measured through spectral analysis. During examination, the patients were in the supine position, with their head rotated 45° to the opposite side of the carotid artery being imaged. Initially, a transverse sweep was performed from the lower neck upward to the carotid artery bifurcation and then into the ICA. The flow velocity and stenosis rate were measured at the site of the common carotid artery, bulb, and proximal ICA. The degree of carotid artery stenosis was estimated in accordance with the European Carotid Surgery Trial by using the following equation: 1 – (narrowest ICA diameter/total lumen diameter at narrowest site) (2, 11, 12). The severity of stenosis was categorized into grades I (0–29%), II (30–49%), III (50–59%), IV (60–69%), V (70–79%), VI (80–89%), VII (90–99%), and VIII (100%) (4).

Stenosis progression was defined as an increase in the degree of stenosis by at least one category. The progression of stenosis in either one or both ICAs was considered as progressive disease. Any change in ICA stenosis in either one or both ICA to a higher grade was considered stenosis progression (13, 14). The patient's stenosis grade was defined on the basis of the stenosis grade of the ICA side with the most severe stenosis.

### Statistical methods

This study involved 179 patients and 358 vessels. Univariate analysis was performed on the basis of λ^2^. An odds ratio was used to measure the association between the grade of stenosis and the risk of stenosis progression. Multivariable logistic regression analysis was used to analyze the risk factors of stenosis progression in the ICA. The results of the logistic regression models are presented as the odds ratio (OR) and the 95% CI. A two-sided *P* < 0.05 was considered as statistically significant. Calculations were performed with SPSS for Windows (version 20.0, SPSS Inc).

## Results

A total of 191 patients were admitted to our hospital for stroke and underwent carotid sonography during hospitalization and at 3 years after stroke onset. Among these 191 patients, eight patients were excluded because the severity of their ICA stenosis exceeded 70%, and two were excluded because the initial severity of their stenosis was less than 70% stenosis but the Follow up carotid sonography showed the patient had received stent implantation. Two patients were excluded because of tortuosity in the carotid artery. The analysis included 179 patients. Patient characteristics are presented in Table 1. The majority 79.9% (143/179) of patients had a history of hypertension; 32.9% (59/179) had pure hypertriglyceridemia; 41.3% (74/179) had hypercholesterolemia; and 29.6% (53/179) had hyper-LDL-c. All of the 179 patients were followed up regularly in the outpatient department of the hospital and regularly took antiplatelet medication (aspirin 100 mg qd, or clopidogrel 75mg qd). Medication for hypertension, diabetes mellitus, and hyperlipidemia were given when the patient had those disease. The physician adjusted statin administration (atorvastatin, Fluvastatin, rosuvstatin, pitavastatin) as necessary with the aim of maintaining a serum cholesterol level lower than 160 mg/dl and an LDL cholesterol level lower than 100 mg/dl. This study included 358 carotid arteries. Among the included vessels, 281, 60, 10, and 7 vessels had stenosis grades of 0–29%, 30–49%, 50–59%, and 60–69%, respectively (Tables 2, 3). Two patients had stenosis with a severity of >50% in both ICA. Univariate analysis revealed that male sex (38.9% vs. 22.5%, *p* = 0.03), smoking (41.9% vs. 25%, *p* < 0.01), hyper-LDL-c (43.4% vs. 25.4%, *p* = 0.02), and stenosis >50% (51.7% vs. 22.7%, *p* < 0.01) increase the risk of stenosis progression. Age, diabetes mellitus, hypertension, hypercholesterolemia, statin use and body mass index (BMI) are not associated with the risk of stenosis progression.

**Table 1:** Characteristics of the study population (*n* = 179).

**Characteristics**	**Total (*n* = 179)**	**Progress (*n* = 55)**	**No progress (*n* = 124)**	*p*-value^*^
Age, mean (SD) years	67.95 ± 11.39	70.74 ± 9.53	66.60 ± 11.99	0.02
**AGE (YEARS)**				
≥65	62	14 (22.6%)	48 (77.4%)	0.06
>65	117	41 (35.1%)	76 (64.9%)
**SEX**				
Women	71	13 (18.3%)	58 (81.7%)	0.03
Men	108	42 (38.9%)	66 (61.1%)
**DIABETES MELLITUS**				
Yes	63	24 (38.1%)	39 (61.9%)	0.08
No	116	31 (26.7%)	85 (73.3%)
**HYPERTENSION**				
Yes	143	47 (32.9%)	96 (67.1%)	0.15
No	36	8 (22.2%)	28 (77.8%)
**HYPER-LDL-C**				
Yes	53	23 (43.4%)	30 (56.6%)
No	126	32 (25.4%)	94 (74.6%)	0.02
**HYPERCHOLESTEROLEMIA**				
Yes	74	28 (37.8%)	46 (62.2%)	0.06
No	105	27 (25.7%)	78 (74.3%)
**STATIN**				
Yes	100	34 (34%)	66 (66%)	0.18
No	79	21 (26.6%)	58 (73.4%)
**SMOKING**				
Yes	83	34 (41%)	49 (59%)	< 0.01
No	96	21 (21.9%)	75 (78.1%)
**STENOSIS**				
0–29%	119	25 (21%)	94 (79%)	< 0.01
30–49%	44	20 (45.5%)	24 (54.5%)
50–59%	9	5 (55.6%)	4 (44.4%)
60–69%	7	5 (74.1%)	2 (28.6%)
**BMI**				
< 24	78	21 (26.9%)	57 (73.1%)	0.52
24–29.9	83	29 (34.9%)	54 (65.1%)
≥30	18	5 (27.8%)	13 (72.2%)

* *χ^2^ test; Progression: patient had ICA stenosis progression at least one grade; Non-progression: patient without ICA stenosis progression; Hyper-LDL-c: patient serum LDL cholesterol level > 130 mg/dl*.

**Table 2:** Rate of carotid artery stenosis and risk of stenosis progression.

Stenosis^*^	**Total**	**Progress**	**OR**	***p*-value**	**95% CI**
0–29%	119	25	1	
30–49%	44	20	3.1	< 0.01	1.49–6.56
50–59%	9	5	4.7	0.03	1.17–18.80
60–69%	7	5	9.4	< 0.01	1.72–51.35

* *Stenosis rate of the side with intensive stenosis*.

**Table 3:** Results of ultrasound examination of the patients' carotid arteries.

**Age (years)**	**Baseline stenosis (grade)**				**Follow up stenosis (grade)**				
	**I**	**II**	**III**	**IV**	**I**	**II**	**III**	**IV**	**>IV**
18–44	5	1	0	0	6	0	0	0	0
45–59	31	6	1	0	26	9	0	2	1
60–79	66	32	8	6	55	41	8	5	3
≥80	17	5	0	1	12	8	2	0	1
Total	119	44	9	7	99	58	10	7	5

*Grade: stenosis grade of the side with intensive stenosis*.

### Carotid stenosis rate and stenosis progression

Among the 358 ICAs from 179 patients, 281 had grade I stenosis, 60 had grade II stenosis, 10 had grade III stenosis, and 7 had grade IV stenosis (Table 4). After 3 years of follow-up, among the 281 vessels with grade I stenosis, 45 had progressed to grade II and two had progressed to grade III. Among the 60 vessels with grade II stenosis, 8 had progressed to grade III, three had progressed to grade IV, one had progressed to grade V, and one had progressed to grade VIII. Among the vessels with grade III stenosis, one and two had progressed to grades IV and V, respectively. Among vessels with grade IV stenosis, one vessel had progressed to grade VI (Table 4). Among the 358 vessels, after 3 years of follow up, 68 showed stenosis progression, among which 11 presented drastic stenosis progression (stenosis progression ≥2 grades).

**Table 4:** Stenosis rate and progression (358 vessels).

**Stenosis**	**Total**	**1 grade**	**2 grade**	**3 grade**	**Over 3 grade**
0–29%	281	45	2	0	0
30–49%	60	8	3	1	1
50–59%	10	1	2	0	0
60–69%	7	0	1	0	0

*Stenosis: stenosis rate of each vessel at baseline, 1 Grade: stenosis progressed to upper one grade, 2 Grade: stenosis progressed to upper second grade, 3 Grade stenosis progressed to upper third grade. Over 3 Grade: stenosis progressed over three grade*.

Patients with stenosis rates of above 50% are at a higher risk of stenosis progression than those with stenosis rates of < 50% (Table 1). Among the 179 included patients, the risk of stenosis progression increased with the severity of stenosis (Table 2). Of the 64 vessels exhibiting stenosis progression, 7 showed stenosis progression on the side with intensive stenosis, 17 showed stenosis progression on the side opposite the side with intensive stenosis, and 22 showed stenosis progression their baseline stenosis rate was in the same grade on both sides of the ICA. Nine patients presented stenosis progression on both sides of the ICA. The mean progression rate was 16.2 ± 10.6% in the 64 vessels with stenosis progression. This result showed that the stenosis progression rate was not higher on the intensive stenosis side.

### Effect of hypercholesterolemia and statin use on the progression of carotid artery stenosis

Hyper-LDL-c increased the risk of ICA stenosis progression but hypercholesterolemia did not. The use of statins did not affect the progression of stenosis in patients with hypercholesterolemia or hyper-LDL-c or in the patients without hypercholesterolemia (Table 5).

**Table 5:** Relationship between statin and the progression of carotid artery.

**Characteristics**	**Statin use**		**Progress**	**Non progress**	*P*-value^*^
Hyperlipidemia *N* = 74	Yes	62	21 (33.9%)	41 (66.1%)	0.19
	No	12	7 (58.3%)	5 (41.7%)
No hyperlipidemia *N* = 105	Yes	38	13 (34.2%)	25 (65.8%)	0.10
	No	67	14 (20.9%)	53 (79.1%)
Hyper-LDL-c *N* = 53	Yes	45	19 (42.2%)	26 (57.8%)	0.71
	No	8	4 (50%)	4 (50%)

* *χ^2^ test, Hyper-LDL-c: patient serum LDL cholesterol level >130 mg/dl*.

Adjustment for confounding factors revealed that hyper-LDL-c increased the risk of stenosis progression, which also increased as the stenosis grade increased. Relative to patients with ICA stenosis < 30%, patients with ICA stenosis >60% were at a higher risk of carotid artery stenosis progression (OR: 6.61, *p* = 0.03) (Table 6).

**Table 6:** Factors that affect the progression of carotid artery stenosis.

**Characteristics**	**Total**	**Progress**	**Odds ratio**	**95% CI**	*P*-value*
**AGE**					
≤ 65	62	14 (22.6%)	1	
> 65	117	41 (35.0%)	1.63	0.72–3.65	0.23
**SEX**					
Men	108	42 (38.9%)	1	
Women	71	13 (18.3%)	0.44	0.16–1.16	0.09
**DIABETES MELLITUS**					
No	117	31 (26.5%)	1	
Yes	62	24 (38.7%)	1.38	0.65–2.94	0.39
**HYPERTENSION**					
No	36	8 (22.2%)	1	
Yes	143	47 (32.9%)	1.66	0.60–4.54	0.32
**SMOKING**					
No	96	21 (21.9%)	1	
Yes	83	34 (41%)	1.39	0.54–3.55	0.48
**Hyper-LDL-C**					
No	126	32 (25.4%)	1	
Yes	53	23 (43.4%)	2.22	1.05–4.71	0.03
**STENOSIS RATE**					
0–29%	119	25 (21%)	1	
30–49%	44	20 (45.5%)	2.33	1.05–5.17	0.03
50–59%	9	5 (55.5%)	3.50	0.81–15.04	0.09
60–69%	7	5 (71.4%)	6.61	1.01–39.61	0.03

* *Logistic regression, Hyper-LDL-c: patient serum LD*.

During the follow-up period, among the 124 patients who did not exhibit ICA stenosis progression, 22 experienced recurrent stroke. Among the 55 patients who exhibited ICA stenosis progression, 11 experienced recurrent stroke. The recurrent stroke rates in the progression and non-progression groups were not significantly different (*p* = 0.39). Of the 11 patients who had ICA stenosis progression and recurrent stroke, two had posterior circulation infarct, four had a stroke on the same side as ICA stenosis progression and five had recurrent stroke on the side opposite stenosis progression. Recurrent stroke appears unrelated to ICA stenosis progression.

## Discussion

We found that stenosis progressed in 17.9% (64/358) of ICA in 30.7% (55/179) of patients. Among the vessels, 15.1% (54/358) progressed by one grade, and 2.8% (10/358) of vessels progressed by two or more grades. Our results for the ICA stenosis progression rate correspond with those reported by Liapis et al., who showed that over 44 months of follow up, 19% of patients exhibited ICA stenosis progression. The incidence of stenosis progression was 24% in the patients who had initial stenosis severity ≥50% and 16% in patients who had initial stenosis severity < 50% (4). We found that the incidence of progression was 16.8% (64/341) in vessels with < 50% stenosis and 23.5% (4/17) in vessels with ≥50% stenosis. Nine patients exhibited bilateral ICA stenosis progression and 46 patients exhibited unilateral ICA stenosis progression.

Fabris et al. reported that diabetes mellitus and total cholesterol are associated with the narrowing of the carotid artery (15). We found that male sex, smoking, and the severity of carotid artery stenosis are associated with the progression of carotid artery atherosclerosis. However, advanced age and hypercholesterolemia do not increase the risk of progression of carotid artery atherosclerosis. Moreover, multiple logistic regression analysis revealed that sex and smoking do not increase the risk of carotid artery stenosis progression; hyper-LDL-c and severity of stenosis associated with carotid artery stenosis progression. Drastically reducing cholesterol levels can reverse the progression of carotid artery atherosclerosis. Furberg et al. showed that lovastatin therapy can regress intima thickening (16). Zhang et al. found that decreasing lower low-density lipoprotein cholesterol levels to < 70 mg/dl can decrease plaque progression (17). We found that hyper-LDL-c increased the risk of stenosis progression but hypercholesterolemia do not affect the progression of carotid artery stenosis, and that statin use does not affect the progression of carotid artery stenosis in patients with and without hypercholesterolemia. The difference in results may be attributed to the inability of our patients to lower their LDL levels to less than 70 mg/dl.

We found that stenosis progression is asymmetric and tends to occur on either side, irrespective of the severity of stenosis. This result corresponds with the lack of association between stenosis progression and hyperlipidemia or statin use because hyperlipidemia and statin use generally affect all vessels, not only one.

Lipais et al. showed that in a population with any degree of asymptomatic stenosis or symptomatic stenosis < 50% (4), age, sex, diabetes mellitus, hypertension, hypercholesterolemia, and smoking habit do not affect stenosis progression and that the overall mean annual stenosis progression rate is 3%. Moreover, 15% of the population will exhibit disease progression. We found that after 3 years of follow-up, the ICA stenosis progression rate was 16.2 ± 10.6%, which is lower than the rate reported by Lipais et al. We also showed that the effects of sex, age, diabetes mellitus, hypertension, hypercholesterolemia, and smoking habit on the progression of stenosis are not statistically significant. The difference in stenosis progression rates between our study and that of Lipais et al. may be related to differences in the characteristics of plaque. Lipais et al. found that echolucent plaques are associated with the increased incidence and rate of progression of stenosis (4). Many complex factors may affect the progression of carotid artery stenosis. Batagini et al. showed that patients with carotid artery progression have higher serum urea and fibrinogen levels than those without carotid artery stenosis progression (18) and those patients with high BMIs have a low risk of stenosis progression. We did not confirm these results. Instead, we found that BMI does not affect the progression of carotid artery stenosis (Table 1). Kamarck et al. reported that daily activity levels are associated with the enhanced progression of carotid artery atherosclerosis (19). Cardiovascular reactivity is a risk factor in atherosclerosis progression. Peter et al. showed that reactivity to psychogenic stress is a predictor of the progression of atherosclerosis (20). Inflammation is also a risk factor of atherosclerosis (21). Schillinger et al. reported that elevated serum high-sensitivity C-reactive protein and serum amyloid A levels are associated with progressive atherosclerosis. Previous studies on carotid artery stenosis have mainly focused on the symptoms of stroke (22, 23).

We found that during 3 years of follow up, 17.9% (64/358) of vessels exhibited ICA stenosis progression. Most of the vessels exhibited ICA progression by one grade, indicating that the severity of stenosis progressed by 10–20%. Moreover, 2.79% (10/358) of the vessels progressed by two or more grades. After 3 years of follow up, eight vessels with mild or moderate stenosis progressed to severe stenosis (≥70%) and were considered potential candidates for endarterectomy or stent (22). No patients with ICA stenosis rates of 0–29% progressed to severe stenosis. However, carotid artery atherosclerosis progression is not uncommon; it was found in 27.6% (45/163) of patients with carotid artery stenosis < 50%. The aim of carotid ultrasound examination is to identify potential candidates for carotid artery stent implantation or endarterectomy (22, 24). We suggest that patients with ICA stenosis rates < 30% be followed up for more than 3 years, whereas those with ICA stenosis rates of 30–49% should be followed up every 3 years through carotid ultrasonography. Patients with stenosis >50% may require annual carotid artery ultrasonography especially in patients who have hyper-LDL-c.

Our study is limited by its retrospective nature. We only investigated patients who were regularly followed up through carotid ultrasonography. Moreover, we did not investigate the characteristics of plaque, which may affect the progression of carotid atherosclerosis.

## Ethics statement

This study was approved by the internal review board (IRB) of the hospital (CYCH-IRB: 096022).

## Author contributions

C-TO substantial contribution to the conception, study design, data analysis, drafting the manuscript and final approval of the version to be published. Y-SW contribution to conception, study design, data acquisition, interpretation and statistical analysis of the data. S-FS substantial contribution to the conception, design, confirm ultrasound data, patient care, revising critical data of the work and final approval of the version to be published. C-SW, Y-CH, Y-HS, and L-CH substantial contribution to the conception, study design, patient care, data collection and check, revision critical data of the work.

### Conflict of interest statement

The authors declare that the research was conducted in the absence of any commercial or financial relationships that could be construed as a potential conflict of interest.

## References

[1] Eastcott HHG Pickering GW Rob CG Reconstruction of internal carotid artery in a patient with intermittent attacks of hemiplegia. Lancet (1954) 264:994–6. 10.1016/S0140-6736(54)90544-9 13213095

[2] European >Carotid Surgery Trialists' Collaborative Group Randomised trial of endarterectomy for recently symptomatic carotid stenosis: final results of the MRC European Carotid Surgery Trial (ECST). Lancet (1998) 351:1379–87. 9593407

[3] Barnett HJM Taylor DW Haynes RB Sackett DL Peerless SJ Ferguson GG. Beneficial effect of carotid endarterectomy in symptomatic patients with high-grade carotid stenosis. N Engl J Med. (1991) 325:445–53. 10.1056/NEJM199108153250701 1852179

[4] Liapis C Kakisis J Papavassiliou V Ntanou A Kontopoulou S Kaperonis E. Internal carotid artery stenosis: rate of progression. Eur J Vasc Endovasc Surg. (2000) 19:111–7. 10.1053/ejvs.1999.0951 10727358

[5] Delcker AM Diener HCM Wilhelm HP. Influence of vascular risk factors for atherosclerotic carotid artery plaque progression. Stroke (1995) 26:2016–22. 10.1161/01.STR.26.11.2016 7482641

[6] Karppi J Kurl S Ronkainen K Kauhanen J Laukkanen JA. Serum carotenoids reduce progression of early atherosclerosis in the carotid artery wall among Eastern Finnish men. PLoS ONE (2013) 8:e64107. 10.1371/journal.pone.0064107 23700460 PMC3660262

[7] Salonen JT Korpela H Salonen R Nyyssonen K. Precision and reproducibility of ultrasonographic measurement of progression of common carotid artery atherosclerosis. Lancet (1993) 341:1158–9. 10.1016/0140-6736(93)93184-3 8097848

[8] Fujishiro K Diez Roux AV Landsbergis P Kaufman JD Korcarz CE Stein JH. Occupational characteristics and the progression of carotid artery intima-media thickness and plaque over 9 years: the Multi-Ethnic Study of Atherosclerosis (MESA). Occup environ Med. (2015) 72:690–8. 10.1136/oemed-2014-102311 25217203 PMC4560665

[9] Jeng JS Chung MY Yip PK Hwang BS Chang YC. Extracranial carotid atherosclerosis and vascular risk factors in different types of ischemic stroke in Taiwan. Stroke (1994) 25:1989–93. 10.1161/01.STR.25.10.1989 8091442

[10] Chang YJ Ryu SJ Chen JR Hu HH Yip PK Chiu TF. [Guidelines for the general management of patients with acute ischemic stroke]. Acta Neurol Taiwan (2008) 17:275–94. 19280874

[11] Mathiesen EB Bønaa KH Joakimsen O. Echolucent plaques are associated with high risk of ischemic cerebrovascular events in carotid stenosis: the Tromsø study. Circulation (2001) 103:2171–5. 10.1161/01.CIR.103.17.2171 11331258

[12] Moneta GL Edwards JM Papanicolaou G Hatsukami T Taylor LM Jr Strandness DE Jr. Screening for asymptomatic internal carotid artery stenosis: duplex criteria for discriminating 60% to 99% stenosis. J Vasc Surg. (1995) 21:989–94. 10.1016/S0741-5214(95)70228-8 7776480

[13] Sutton-Tyrrell K Wolfson SK Kuller LH. Blood pressure treatment slows the progression of carotid stenosis in patients with isolated systolic hypertension. Stroke (1994) 25:44–50. 10.1161/01.STR.25.1.44 8266382

[14] Sabeti S Exner M Mlekusch W Amighi J Quehenberger P Rumpold H Prognostic Impact of Fibrinogen in Carotid Atherosclerosis: nonspecific indicator of inflammation or independent predictor of disease progression? Stroke (2005) 36:1400–4. 10.1161/01.STR.0000169931.96670.fc 15933258

[15] Fabris F Zanocchi M Bo M Fonte G Poli L Bergoglio I. Carotid plaque, aging, and risk factors a study of 457 subjects. Stroke (1994) 25:1133–40. 10.1161/01.STR.25.6.1133 8202970

[16] Furberg CD Adams HP Jr Applegate WB Byington RP Espeland MA Hartwell T. Effect of lovastatin on early carotid atherosclerosis and cardiovascular events. Asymptomatic Carotid Artery Progression Study (ACAPS) Research Group. Circulation (1994) 90:1679–87. 10.1161/01.CIR.90.4.1679 7734010

[17] Zhang Q Liu S Liu Y Hua Y Song H Ren Y. Achieving low density lipoprotein-cholesterol < 70mg/dL may be associated with a trend of reduced progression of carotid artery atherosclerosis in ischemic stroke patients. J Neurol Sci. (2017) 378:26–9. 10.1016/j.jns.2017.04.024 28566171 PMC5802361

[18] Batagini NC da Silva ES Pinto CA Puech-Leao P de Luccia N. Analysis of risk factors and diseases associated with atherosclerosis in the progression of carotid artery stenosis. Vascular (2016) 24:59–63. 10.1177/1708538115571404 25687720

[19] Kamarck TW Shiffman S Sutton-Tyrrell K Muldoon MF Tepper P. Daily psychological demands are associated with 6-year progression of carotid artery atherosclerosis: the Pittsburgh Healthy Heart Project. Psychosom Med. (2012) 74:432–9. 10.1097/PSY.0b013e3182572599 22582340 PMC4869071

[20] Barnett PA Spence JD Manuck SB Jennings JR. Psychological stress and the progression of carotid artery disease. J Hypertens. (1997) 15:49–55. 10.1097/00004872-199715010-00004 9050970

[21] Schillinger M Exner M Mlekusch W Sabeti S Amighi J Nikowitsch R. Inflammation and Carotid Artery–Risk for Atherosclerosis Study (ICARAS). Circulation (2005) 111:2203–9. 10.1161/01.CIR.0000163569.97918.C0 15851593

[22] Turkenburg JL van Oostayen JA Bollen WL. Role of carotid sonography as a first examination in the evaluation of patients with transient ischemic attacks and strokes: benefit in relation to age. J Clin Ultrasound (1999) 27:65–9. 9932250 10.1002/(sici)1097-0096(199902)27:2<65::aid-jcu3>3.0.co;2-m

[23] Walker MD Marler JR Goldstein M Grady PA Toole JF Baker WH Endarterectomy for asymptomatic carotid artery stenosis. JAMA (1995) 273:1421−8. 10.1001/jama.1995.035204200370357723155

[24] Karlsson L Kangefjard E Hermansson S Strömberg S Österberg K Nordanstig A. Risk of recurrent stroke in patients with symptomatic mild (20–49% NASCET) carotid artery stenosis. Eur J Vasc Endovasc Surg. (2016) 52:287–94. 10.1016/j.ejvs.2016.05.014 27369293

